# Comparison of three cryotherapy techniques for early post-TKA pain control in terms of efficacy and patient satisfaction: a randomized controlled trial

**DOI:** 10.1186/s42836-024-00287-7

**Published:** 2025-01-08

**Authors:** Keerati Chareancholvanich, Worawut Keesukpunt, Chaturong Pornrattanamaneewong, Rapeepat Narkbunnam, Atthakorn Jarusriwanna

**Affiliations:** 1 Department of Orthopaedic Surgery, Faculty of Medicine Siriraj Hospital, Mahidol University, 2 Wang Lang Road, Siriraj, Bangkok Noi, Bangkok, 10700 Thailand; 2 Bone and Joint Center, Thonburi Hospital, 34/1 Soi Itsaraphap 44, Ban Chang Lo, Bangkok Noi, Bangkok, 10700 Thailand; 3 Department of Orthopaedics, Faculty of Medicine, Naresuan University, 99 Moo 9, Phitsanulok-Nakhon Sawan Road, Tha Pho, Mueang Phitsanulok, Phitsanulok, 65000 Thailand

**Keywords:** Total knee arthroplasty, Knee osteoarthritis, Multimodal analgesia, Cryotherapy, Cold pack, Cryo-cuff

## Abstract

**Background:**

Cryotherapy is a non-pharmacological option that complements drug therapy to achieve the most comprehensive multimodal analgesia. Various techniques are currently available, including the conventional gel cold pack, the cryo-cuff, and a novel mobile cold compression device (MCCD). This study aimed to evaluate and compare three cryotherapy techniques in terms of efficacy and patient satisfaction in patients undergoing total knee arthroplasty (TKA).

**Methods:**

This prospective randomized study included 108 patients who were scheduled for primary unilateral TKA. The patients were allocated to 3 groups for postoperative cryotherapy techniques: gel cold pack, cryo-cuff, and MCCD. Scores on the visual analog scale (VAS) for pain intensity, morphine consumption, knee range of motion (ROM), knee swelling, length of hospital stay, and patient satisfaction were collected.

**Results:**

Postoperative VAS scores showed a significant difference among the groups at 8 and 72 h after surgery (*P* = 0.002 and 0.026, respectively). At the earliest postoperative time point, post hoc analysis demonstrated that patients in the MCCD group had lower pain scores than those in the gel cold pack (*P* < 0.001) and the cryo-cuff group (*P* = 0.030). However, cryo-cuff reduced knee swelling significantly compared to gel cold pack (*P* = 0.028) and MCCD (*P* = 0.011) at postoperative 72 h. The total satisfaction score was 86.8, 82.8, and 89.1 with gel cold pack, cryo-cuff, and MCCD, respectively.

**Conclusions:**

Cryotherapy is an adjunct to post-TKA pain control at the surgical site. MCCD has shown superior efficacy in pain reduction during the earliest postoperative period, and achieved high patient satisfaction.

**Trial registration:**

This study was registered in the Thai Clinical Trials Registry database (no. TCTR20200517002).

## Background

Pain control after total knee arthroplasty (TKA) is essential to improved rehabilitation, better functional outcomes, and high patient satisfaction. About 15%–30% of patients experienced persistent postoperative pain up to 12 months after surgical intervention [1, 2]. The efficacy of acute postoperative pain control, especially in the first 72 h after surgical intervention, is a key contributor to the persistence of postoperative pain. Poor pain control during this period has been found to be a risk factor for chronic postsurgical pain, which is associated with subsequent inferior functional outcomes and low patient satisfaction lasting for up to 2 years [3, 4].

Multimodal analgesia, which includes the use of several analgesic medications, regional anesthesia techniques, and non-pharmacological strategies, has been proven to minimize postoperative pain in patients undergoing TKA. This method targets various pain pathways and acts synergistically to enhance effect of pain control and, thus, early recovery of patients [5]. Cryotherapy is the use of non-pharmacological cold substances at areas surrounding surgical sites, which theoretically induces vasoconstriction, diminishes nerve conduction velocity, and decreases edema. Various cryotherapy techniques have been introduced and widely used to enhance recovery, minimize pain, and reduce swelling. Traditional gel cold packs and other cryotherapy devices, *e.g*., continuous cold flow cryotherapy, dynamic intermittent compression cryotherapy, and computer-assisted cryotherapy, have been shown to be effective in previous studies [6–9]. However, conventional gel cold packs often have limitations in terms of coverage area, which may not conform well to movable body parts, especially knee joints. In contrast, the newer technology comes with an additional cost for the devices. We introduced a novel mobile cold compression device (MCCD), a locally made mobile instrument comprised of a simple socket with a maximum of 3 gel cold packs attached all together but able to fold between each gel cold pack. This device is applied to the knee joint and allows patients to flex and extend the knee during rehabilitation.

The purpose of this study was to evaluate and compare three cryotherapy techniques, the gel cold pack, the cryo-cuff, and the MCCD, in terms of efficacy and satisfaction in patients undergoing primary unilateral TKA. The hypothesis asserted that the MCCD, which offers a greater coverage area and allows for more mobility during ambulation, would attain better pain reduction and greater patient satisfaction compared to other techniques.

## Methods

The protocol for this randomized controlled study was approved by the Institutional Review Board. Written informed consent was obtained from all study participants before allocation. Included in this study were patients aged > 50 years who were diagnosed with tricompartmental knee osteoarthritis, fulfilling the radiological criteria of the Kellgren-Lawrence Classification grade 3–4 [10], and scheduled for primary unilateral TKA were included in this study. Exclusion criteria were: (1) having received previous surgeries involving the index knee, (2) having history of knee infection, (3) being diagnosed with secondary knee osteoarthritis, (4) being allergic to any medications included in the study's regimen, and (5) having any conditions that were potentially dangerous with cryotherapy (cold urticaria, diabetes, deep vein thrombosis, and peripheral vascular diseases).

### Surgical procedure, randomization, and outcome measurement

**Fig. 1:**
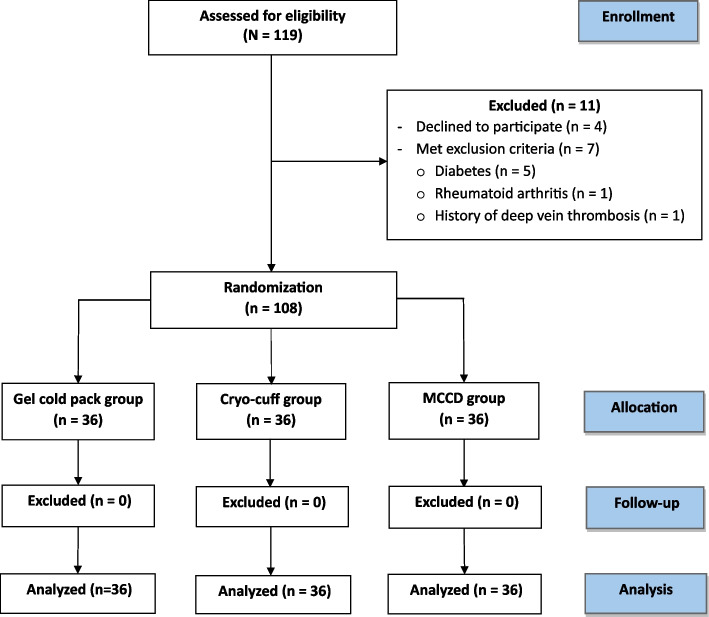
Consolidated Standards of Reporting Trials (CONSORT) diagram showing the flow of patients in the study

A total of 119 candidate patients who underwent unilateral primary TKA were enrolled. Eleven patients were excluded for the following reasons: 5 had underlying diabetes, 4 declined to participate in the study, 1 had rheumatoid arthritis, and 1 had a history of deep vein thrombosis. The remaining 108 patients were included for group assignment (Fig. 1). All patients received the identical preemptive medication regimen with gabapentin on the night before the index surgery. Additionally, acetaminophen and celecoxib were administered to all patients 2 h prior to surgery. All surgical procedures were performed by a single surgeon under spinal anesthesia with bupivacaine. Furthermore, the adductor canal block was performed by an anesthesiologist before operative procedures. A uniform tourniquet inflation pressure of 300 mmHg was applied prior to skin incision and released immediately after skin closure. Fixed-bearing cemented posterior-stabilized (PS) TKA (NexGen® LPS; Zimmer Biomet, Warsaw, IN, USA) was implanted in all patients via a medial parapatellar approach. Before the arthrotomy closure, a deep suction drain was applied and then removed within 48 h after surgery. The postoperative protocol that included a non-compressive wound dressing, physical therapy, and early ambulation with a walker was encouraged for all patients [11]. Intravenous 40 mg of parecoxib was administered every 12 h, immediately after surgery to the postoperative 48 h. Additional intravenous 3 mg of morphine sulfate was injected as needed for breakthrough pain every 4 h if the 10 cm visual analog scale (VAS) score was above 5.

**Fig. 2:**
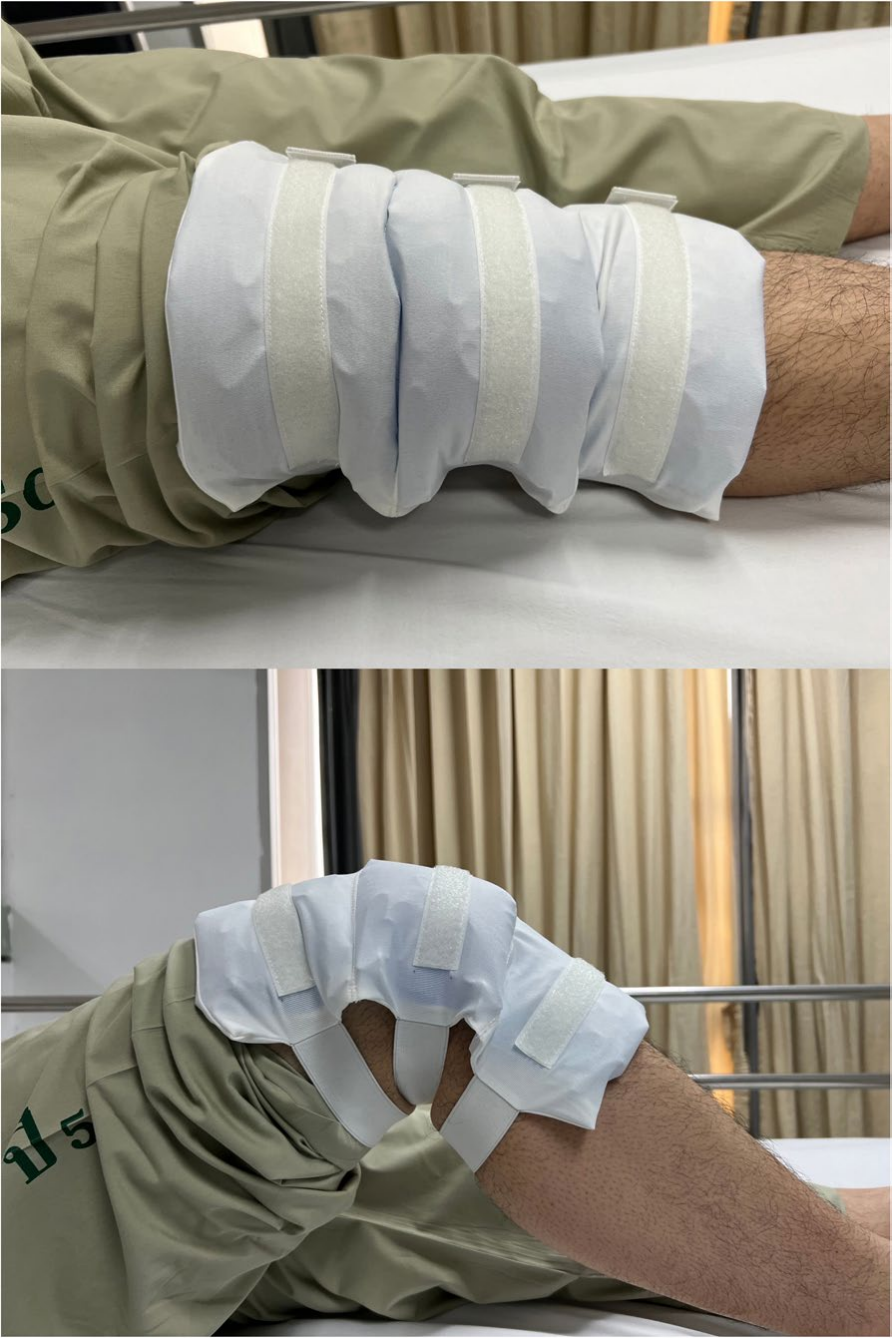
A mobile cold compression device (MCCD) during knee extension (above) and knee flexion (below)

Randomization with a computer-generated sequential block-of-six technique was performed before surgical intervention to determine postoperative cryotherapy methods. Patients in the gel cold pack group received 4 inch × 10 inch (10.2 cm × 25.4 cm) conventional gel cold pack (Nexcare™, St. Paul, MN, USA) that had been conserved at −17ºC, while patients in the cryo-cuff group received a device including a motorized pneumatic pump cooler with a tube connecting to a circumferential knee pad sized 10 inch × 19 inch (25.4 cm × 48.3 cm) (Aircast®, Lewisville, TX, USA), and patients in the MCCD group received a device which comprised of a foldable socket and 3 gel cold packs (Fig. 2). All devices were applied and wrapped anteriorly onto the operated knee at mid-patella level immediately after surgery and replaced with reusable colder devices (or refilled with ice in the cryo-cuff group) every 6 h until the patients were discharged from the hospital. Then all patients were assigned for routine follow-up at 2 weeks postoperatively.

Demographic data, including age, sex, body mass index (BMI), side of operated knee, operative time, and preoperative functional outcomes evaluated by the Oxford Knee Score (OKS), were recorded. The OKS is a patient-reported outcome measure specifically designed to assess the function and pain associated with knee osteoarthritis. It consists of 12 questions covering different aspects of knee function and the impact of knee problems on daily life. Each question is scored on a scale, with lower total scores indicating more severe symptoms [12].

The primary outcome of this study was the postoperative pain intensity evaluated by the 10 cm VAS. Patients were evaluated for the maximum pain at rest every 8 h for the first 72 h and again 2 weeks after surgery. The other outcomes included total morphine use at 24, 48, and 72 h after surgical intervention, the length of hospital stay, and knee function in terms of the maximum angle of knee flexion and extension lag measured by a universal long-arm goniometer at 72 h and 2 weeks after surgery. The knee flexion angle was measured when the patient was lying down in a supine position and then asked to actively flex the knee [13]. The extension lag was evaluated by measuring the remaining angles in which the patient was unable to actively extend the knee in a sitting position [14]. Furthermore, swelling of the knee joint was assessed by girth measurement 72 h and 2 weeks after surgery, compared to preoperative baseline measurement. The patient was placed in a supine position with full knee extension and then the circumference was measured at 10 cm proximal to the superior pole of the patella [15]. All the following outcomes were recorded by the group of assessors who were blinded to the cryotherapy techniques.

The satisfaction with the cryotherapy techniques was evaluated before patients were discharged to home in terms of 4 primary aspects: pain relief, convenience, comfort, and overall satisfaction. Measurement was modified from the Self-Administered Patient Satisfaction Scale (SAPS) proposed by Mahomed et al. [16] Each domain is rated, on a four-point Likert scale, as “very satisfied”, “somewhat satisfied”, “somewhat dissatisfied”, and “very dissatisfied”, which corresponded to scores of 100, 75, 50, and 25, respectively. The total satisfaction score was then calculated as the mean of the scores of the individual domains, ranging from 25 to 100 (Higher scores are indicative of greater satisfaction.).

### Data analyses

All characteristics and measured outcomes were presented as numbers and percentages for categorical variables and mean and standard deviation (SD) for continuous variables. The normality of the data was assessed using the Kolmogorov–Smirnov test. The comparison among groups was made using analysis of variance (ANOVA) and the post hoc pairwise comparison was performed using the Bonferroni test after a significant difference was found. The sample size for this study was calculated using the formula for comparing two independent means, and the result showed that at least 32 patients per group were needed to achieve a 80% statistical power to detect a difference of VAS pain score of 1.5 with a significance level of 0.05 [17]. The SD of 1.8 used in the formula was derived from the study by Ruffilli et al. [18], which evaluated the pain management efficacy between a continuous cold flow device and crushed ice packs in TKA patients. However, to account for an anticipated 10% dropout rate, the target enrollment was increased to 36 patients per group. All statistical analyses were conducted using PASW Statistics software (SPSS Inc., Chicago, IL, USA), with statistical significance set at a *P*-value of < 0.05.

## Results

**Fig. 3:**
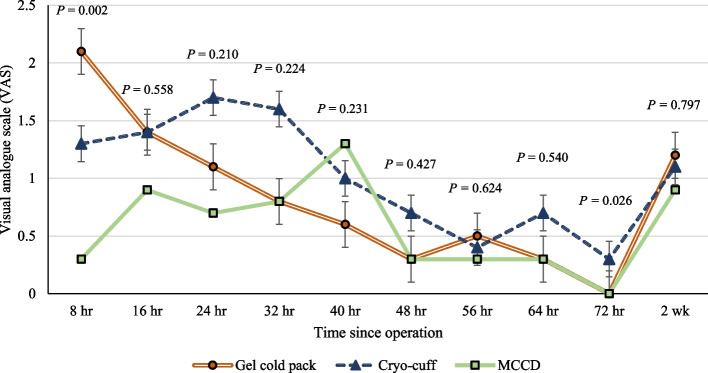
A graph showing the mean postoperative pain VAS score among the 3 groups of cryotherapy techniques. The *P*-value < 0.05 indicates statistical significance among the groups (ANOVA)

**Fig. 4:**
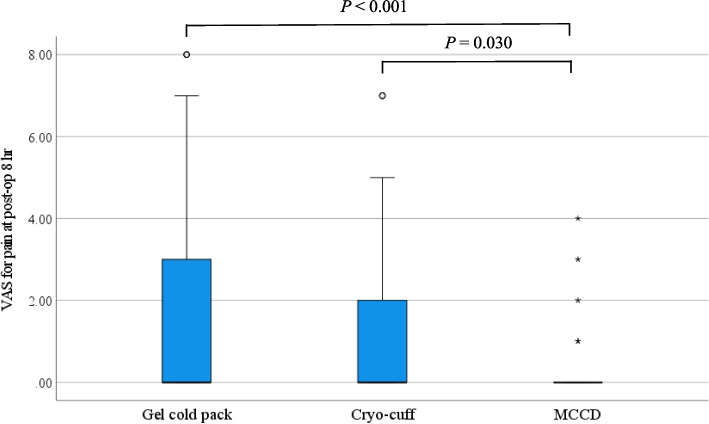
A box plot showing the distribution of the VAS pain score at postoperative 8 h among the 3 groups of cryotherapy techniques. The boxes represent the median, the interquartile range, and the whiskers of the data range. The circles (o) represent the potential outliers and the asterisks (*) display the extreme values. The *P*-value < 0.05 indicates statistical significance

Of the final 108 participants included, 36 patients were assigned to each group after randomization (Fig. 1). Baseline characteristics did not show significant differences among the three groups (Table 1). After surgical intervention and application of cryotherapy devices, the VAS pain score demonstrated a statistically significant difference at 8 and 72 h after surgery, while the other points of time did not show significant differences (Fig. 3). Post hoc pairwise comparisons showed a significant reduction in pain in the MCCD group compared to the gel cold pack and the cryo-cuff groups at 8 h after surgery (*P* < 0.001 for the MCCD vs. gel cold pack group, and *P* = 0.030 for the MCCD vs. cryo-cuff group) (Fig. 4). However, the patients in the cryo-cuff group scored a significantly higher VAS pain score than the gel cold pack and the MCCD group at 72 h after surgery (*P* = 0.018 for both comparisons). The patients in the MCCD group appeared to consume less morphine, but there were no statistically significant differences among the groups in all time frames. The other results, including the maximum knee flexion angle, extension lag, and length of hospital stay, did not show significant differences among the three groups at all time points (Table 2).

**Table 1:** Baseline patients’ demographics

Characteristics	Gel cold pack (*n* = 36)	Cryo-cuff (*n* = 36)	MCCD (*n* = 36)	*P*-value
Age (years)	71.0 ± 1.2	71.0 ± 1.7	70.9 ± 1.2	0.927
Sex				0.797
Female	31 (86.1%)	32 (88.9%)	31 (86.1%)	
Male	5 (13.9%)	4 (11.1%)	5 (13.9%)	
BMI (kg/m^2^)	27.6 ± 0.9	26.8 ± 0.8	28.4 ± 0.8	0.369
Operated side				0.898
Left	19 (52.8%)	17 (47.2%)	20 (55.6%)	
Right	17 (47.2%)	19 (52.8%)	16 (44.4%)	
Operative time (minutes)	71.3 ± 3.8	77.3 ± 3.8	69.4 ± 2.3	0.231
OKS	27.4 ± 1.6	27.0 ± 1.3	26.1 ± 1.3	0.821

Data are presented as mean ± SD for age, BMI, operative time, and OKS

A *P*-value < 0.05 indicates statistical significance among the groups (ANOVA)

**Table 2:** Postoperative outcome parameters compared among cryotherapy groups

Characteristics	Gel cold pack (*n* = 36)	Cryo-cuff (*n* = 36)	MCCD (*n* = 36)	*P*-value
Morphine use (mg)				
24 h	3.0 ± 3.7	2.0 ± 0.5	1.6 ± 1.5	0.110
48 h	2.0 ± 2.7	1.6 ± 1.8	1.5 ± 1.9	0.579
2 h	0.9 ± 1.3	1.0 ± 2.4	0.5 ± 1.0	0.607
The maximum knee flexion angle (degrees)				
72 h	82.7 ± 15.0	77.0 ± 21.2	87.2 ± 17.5	0.182
2 weeks	96.1 ± 11.9	96.3 ± 12.5	101.6 ± 11.2	0.106
Extension lag (degrees)				
72 h	3.3 ± 3.2	3.1 ± 4.0	3.2 ± 3.2	0.927
2 weeks	3.5 ± 2.9	4.6 ± 5.0	2.5 ± 2.5	0.066
Length of hospital stay (days)	3.9 ± 1.1	4.0 ± 0.8	3.8 ± 0.9	0.783
Postoperative knee swelling (cm)				
72 h	3.0 ± 2.0	1.9 ± 1.4	3.1 ± 1.6	0.007
2 weeks	1.6 ± 1.9	0.9 ± 1.8	2.0 ± 1.9	0.063

Data are presented as mean ± SD

A *P*-value < 0.05 indicates statistical significance among the groups (ANOVA)

**Fig. 5:**
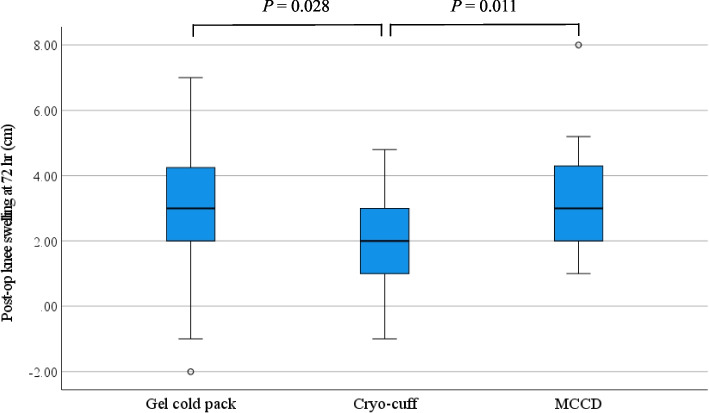
A box plot showing the distribution of knee swelling at postoperative 72 h among the 3 groups of cryotherapy techniques. The boxes represent the median, the interquartile range, and the whiskers of the data range. The circles (o) represent the potential outliers. The *P*-value < 0.05 indicates statistical significance

The reduction of knee swelling was statistically significant 72 h after surgery. The cryo-cuff group knee girth was, on average, 1.9 cm greater, while the girth was averagely 3 and 3.1 cm larger in the gel cold pack and the MCCD group, respectively (Table 2). Post hoc analysis showed a significant difference between the cryo-cuff and gel cold pack group (*P* = 0.028), and the cryo-cuff and the MCCD group (*P* = 0.011) (Fig. 5). However, no statistical significance was found among the three groups two weeks postoperatively.

**Fig. 6:**
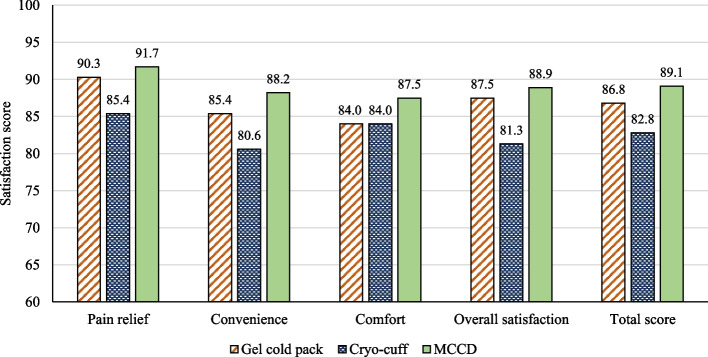
A graph showing the satisfaction scores from patient surveys in 4 aspects (pain relief, convenience, comfort, and overall satisfaction) and a total satisfaction score calculated from the mean of the individual domains in the 3 groups of cryotherapy techniques

The survey demonstrated that patients in the gel cold pack group were satisfied with this technique, with scores for pain relief, convenience, comfort, and overall satisfaction being 90.3, 85.4, 84.0, and 87.5, respectively. Cryo-cuff group yielded a satisfaction score of 85.4, 80.6, 84.0, and 81.3 for pain relief, convenience, comfort, and overall satisfaction, respectively, and the patients in the MCCD group scored 91.7, 88.2, 87.5, and 88.9 for satisfaction in pain relief, convenience, comfort, and overall satisfaction, respectively. The total satisfaction score for gel cold pack, a cryo-cuff, and MCCD groups was 86.8, 82.8, and 89.1, respectively (Fig. 6).

## Discussion

This study revealed that a new MCCD, a cryotherapy technique used as an adjunct was efficacious for early postoperative pain control after TKA. The results demonstrated that the MCCD could lower the VAS pain score significantly at the earliest 8 h after surgical intervention. With regard to patient satisfaction, the MCCD scored higher than the gel cold pack and cryo-cuff in all domains. These outcomes support our theoretical principle that a greater coverage area and more mobility during ambulation would result in superior pain reduction and greater patient satisfaction.

Approximately 10 − 20% of patients were dissatisfied with results after TKA, and in these patients, postoperative pain and limited knee function are the key contributors to their unmet expectations [19, 20]. Several strategies were introduced to minimize these undesirable clinical complications, including pharmacological and non-pharmacological treatments. Cryotherapy is one of the simplest procedures to improve acute postoperative pain management and speed up functional recovery, as cold and compression are generally applied after soft tissue injuries to relieve pain and mitigate swelling [21]. A recent systematic review by Wyatt et al. [22] showed that cryotherapy could decrease opioid consumption and alleviate pain during the first postoperative week, but pain ratings did not achieve a minimal clinically important difference (MCID). Traditional gel cold packs have advantages for low cost, convenience, and easy availability. In previous studies, ice packs were shown to have an efficacy similar to advanced cryotherapy techniques in pain reduction, swelling, postoperative ROM, and blood loss [23, 24]. Nevertheless, the limitation of this device is the area coverage, which might need multiple items, or a larger size compared with the other techniques to provide an equivalent total area coverage. The cryo-cuff provided greater coverage to the knee joint due to its knee pad design. Another advantage of this device is the continuous flow of cold water from the motorized pneumatic pump cooler, which produces a persistent cooling effect and compression over time [25, 26]. However, this instrument requires gravity to fill and empty the knee cuff, so the cooler must be placed higher than the knee joint, but not more than 15 inches (38 cm) to avoid excessive hydrostatic pressure [27]. The size and static position of the cooler can be difficult for patients during walking exercises and the cost of the device is nearly 10 times that of the conventional gel cold pack. A previous study by Schinsky et al. [28] showed that the ice/gel pack cryotherapy had advantage over the circulating cold water device in terms of cost-effectiveness, and the conventional gel cold pack could save US$97.34 per patient receiving TKA.

The MCCD addressed these challenges by incorporating multiple gel cold packs into a socket designed for continuity and foldability, ensuring seamless integration among the gel packs. Patients could use this device during physical therapy, ROM exercise, and walking. Our study found that MCCD accomplished superior efficacy in pain reduction among cryotherapy techniques during the first 24 h after surgery, with a statistically significant difference in the 8 h after surgery. Furthermore, the difference in the mean VAS pain score between patients in the MCCD group and the gel cold pack group at the initial assessment time point reached the MCID of 1.5 for VAS after TKA [17]. However, patients receiving all cryotherapy techniques had a rebound pain intensity at 2 weeks after TKA, which could be sequela after discarding the cryotherapy devices. The resumption of cryotherapy up to this period could be beneficial in terms of pain reduction.

The cryo-cuff was superior in swelling reduction, especially within 72 h after surgery. The reduction in swelling, when compared to both the MCCD and gel cold pack groups, exceeded the MCID of 1.0 cm for knee swelling after TKA [29]. Compression with a continuous flow of cold water by the device might result in a gradual effect of vasoconstriction and hyperemia reduction in surrounding tissues. A comprehensive review by Kunkle et al. [30] revealed that continuous cryotherapy devices had some beneficial effects in swelling reduction compared to bagged ice or ice pack therapy. Another systematic review by Lee et al. [31] showed that the application time per cryotherapy session of an automated device was generally longer than that of an ice pack, which consequently influenced its efficacy. However, our results demonstrated that the reduction in swelling did not necessarily correlate directly with pain relief, as pain is a multifactorial experience influenced by more than just inflammation [32]. Furthermore, while the VAS pain score for the cryo-cuff group was significantly higher compared to the other two methods at the same time point of the swelling reduction, the mean differences in VAS scores between the cryo-cuff and MCCD groups, as well as between the cryo-cuff and gel cold pack groups did not meet the MCID for VAS pain score after TKA.

The patient satisfaction survey in this study was carried out by using SAPS, which was validated in patients with primary total joint arthroplasty, with internal reliability ranging from 0.86 to 0.92 when measured by Cronbach’s alpha [16]. All cryotherapy techniques yielded excellent scores in all domains, and patients in the MCCD group scored higher as compared to others. However, some patients in the cryo-cuff group mentioned that this cryotherapy method had some weaknesses in convenience, such as requiring a static position of the cooler. Meanwhile, the comfort of using this device was similar to that of the conventional gel cold pack.

The clinical relevance of this study lies in its findings regarding the efficacy of using MCCD as an adjunct for early postoperative pain control after TKA. Incorporating MCCD into postoperative care protocols could potentially improve early outcomes post-TKA. Specifically, its ability to provide greater coverage area and enhanced mobility during ambulation may contribute to better pain management and overall patient experience.

This study is subject to some limitations. First, the majority of the subjects in the study were female, which might be a factor affecting postoperative pain and functional outcomes [33]. However, several studies demonstrated that gender did not influence pain intensity and functional recovery after TKA [34, 35]. Second, the duration between each cryotherapy session was quite long, as replacing the gel cold pack/MCCD devices or refilling the ice in the cryo-cuff cooler every 6 h could alter the actual efficacy of the cryotherapy devices. The frequent change of devices or the refilling of ice, when their effectiveness decreased, would represent the precise effect. Third, no patients without receiving cryotherapy served as a control group in this study because the use of the gel cold pack is a standard postoperative protocol in our institution, as its efficacy and cost-effectiveness that we discussed earlier [22, 27]. However, the absence of a non-cryotherapy group may limit the ability to establish a baseline for comparison, making it challenging to determine whether the observed outcomes are attributable to the cryotherapy intervention or simply reflect natural healing or placebo effects. Fourth, the outcome evaluation at 2 weeks postoperatively might be too early to determine the difference among the cryotherapy techniques, a longer follow-up measurement period is needed in future. Finally, the extended duration of cryotherapy use is a point of interest for further studies, but the compliance evaluation when patients return home is a challenge.

## Conclusions

The topical use of cryotherapy is an effective strategy to reduce pain and swelling, with rapid recovery in patients receiving TKA. Utilizing MCCD resulted in superior pain reduction during the earliest postoperative period, with high levels of patient satisfaction reported in terms of pain relief, convenience, comfort, and overall experience throughout the early recovery period.

## Data Availability

The datasets used and/or analysed during the current study are available from the corresponding author on reasonable request.

## Abbreviations

ANOVA: Analysis of variance
BMI: Body mass index
MCCD: Mobile cold compression device
MCID: Minimal clinically important difference
OKS: Oxford Knee Score
PS: Posterior-stabilized
ROM: Range of motion
SAPS: The Self-Administered Patient Satisfaction Scale
SD: Standard deviation
TKA: Total knee arthroplasty
VAS: Visual analog scale
